# Type 2 diabetes, socioeconomic status and life expectancy in Scotland (2012–2014): a population-based observational study

**DOI:** 10.1007/s00125-017-4478-x

**Published:** 2017-10-26

**Authors:** Jeremy Walker, Helen Colhoun, Shona Livingstone, Rory McCrimmon, John Petrie, Naveed Sattar, Sarah Wild, on behalf of the Scottish Diabetes Research Network Epidemiology Group

**Affiliations:** 1 Usher Institute of Population Health Sciences and Informatics, The University of Edinburgh, Medical School, Teviot Place, Edinburgh, EH8 9AG UK; 2 Institute of Genetics and Molecular Medicine, The University of Edinburgh, Edinburgh, UK; 3 Division of Population Health Sciences, University of Dundee, Dundee, UK; 4 Division of Cardiovascular and Diabetes Medicine, University of Dundee, Dundee, UK; 5 Institute of Cardiovascular and Medical Sciences, University of Glasgow, Glasgow, UK

**Keywords:** Epidemiology, Life expectancy, Socioeconomic status, Type 2 diabetes

## Abstract

**Aims/hypothesis:**

The aim of this study was to assess the role of socioeconomic status (SES) in the associations between type 2 diabetes and life expectancy in a complete national population.

**Methods:**

An observational population-based cohort study was performed using the Scottish Care Information – Diabetes database. Age-specific life expectancy (stratified by SES) was calculated for all individuals with type 2 diabetes in the age range 40–89 during the period 2012–2014, and for the remaining population of Scotland aged 40–89 without type 2 diabetes. Differences in life expectancy between the two groups were calculated.

**Results:**

Results were based on 272,597 individuals with type 2 diabetes and 2.75 million people without type 2 diabetes (total for 2013, the middle calendar year of the study period). With the exception of deprived men aged 80–89, life expectancy in people with type 2 diabetes was significantly reduced (relative to the type 2 diabetes-free population) at all ages and levels of SES. Differences in life expectancy ranged from −5.5 years (95% CI −6.2, −4.8) for women aged 40–44 in the second most-deprived quintile of SES, to 0.1 years (95% CI −0.2, 0.4) for men aged 85–89 in the most-deprived quintile of SES. Observed life-expectancy deficits in those with type 2 diabetes were generally greater in women than in men.

**Conclusions/interpretation:**

Type 2 diabetes is associated with reduced life expectancy at almost all ages and levels of SES. Elimination of life-expectancy deficits in individuals with type 2 diabetes will require prevention and management strategies targeted at all social strata (not just deprived groups).

**Electronic supplementary material:**

The online version of this article (10.1007/s00125-017-4478-x) contains peer-reviewed but unedited supplementary material, which is available to authorised users.

## Introduction

The elevation of mortality risk associated with diabetes is well established [1, 2]; one meta-analysis has concluded that mortality in diabetic populations is approximately double that observed in those without diabetes [3], while a Scottish study found that the relative risk of mortality associated with type 2 diabetes compared with a diabetes-free population was 1.35 for men and 1.62 for women [4]. A number of studies have expressed the excess risk of death associated with diabetes in terms of differential life expectancy [5–14]; that is, the mean number of expected additional years of life at specific ages under the assumption that current observed mortality rates remain unchanged throughout the lives of the population of interest. However, few investigations have focused exclusively on type 2 diabetes; rather, studies have tended either not to differentiate between type 1 and type 2 disease [5, 7–9, 13], or to concentrate exclusively on the former [10–12]. A further limitation of work in this area is that although the association between diabetes and mortality appears to be modified by socioeconomic status (SES) [4], and life expectancy in general populations has been found to vary by SES [15, 16], few studies investigating relationships between diabetes and life expectancy have explicitly sought to assess how these relationships are linked to SES. Rather, SES (where it is considered at all) has generally been treated as a potential confounder which may obscure associations between diabetes and the expectation of life. One exception to this is work reported by Perna et al [17], which found that SES-related differences in life expectancy increased when diabetes was present. Because the prevalence of diabetes is higher in deprived groups [18, 19], and mortality risk within diabetic populations (as in general populations) is known to vary by SES [4, 20–22], it is important to estimate the role (not necessarily causal) of SES in the observed associations between the presence of diabetes and life expectancy. Understanding that role will help to clarify whether apparent differences in life expectancy between groups with and without diabetes wholly or partly reflect varying patterns of SES rather than differential diabetes status. The study reported here examined the relationships among these three factors, diabetes, SES, and life expectancy, with two objectives, namely (1) to ascertain whether differences in life expectancy associated with type 2 diabetes are present at all levels of SES and to quantify these differences; and (2) to determine whether patterns of SES-related differences in life expectancy among people with type 2 diabetes are similar to those observed in a general population.

## Methods

### Data sources and study population

The overall design of the study was based on deriving period life tables for (1) a cohort of people with type 2 diabetes, and (2) the remainder of the population of Scotland that does not have type 2 diabetes, permitting the respective life expectations of these two groups to be compared. To create the type 2 diabetes cohort, all individuals living with type 2 diabetes in Scotland at any point during the period 1 January 2012 to 31 December 2014 were identified from the Scottish Care Information – Diabetes (SCI-Diabetes) dataset [23]. SCI-Diabetes is a national register of people with diabetes who are resident in Scotland. Initially developed as a system to aid clinical management of individuals with diabetes, SCI-Diabetes has been extensively used in support of diabetes-related epidemiological research (with appropriate controls to protect patient confidentiality and privacy). The study reported here used data extracted from SCI-Diabetes in 2016. SCI-Diabetes data are available from the Health Informatics Centre at the University of Dundee but restrictions apply to the availability of these data, which were used under license for the current study, and so are not publicly available. Data are, however, available with permission of the Public Benefit and Privacy Panel for Health and Social Care of National Health Service National Services Scotland (NHSNSS). The SCI-Diabetes data were linked to national mortality records by the Information Services Division (ISD) of NHSNSS, thus allowing the vital status of individuals included in the study to be determined throughout the study period. Generation of the linked dataset was approved by the responsible ethics committee, Caldicott guardians and the NHSNSS Privacy Application Committee (application no. 33/11). The data were anonymised prior to the researchers being granted access.

### Statistical analysis

Numbers of person-years and of deaths contributed by the cohort of individuals with type 2 diabetes during the three-year study period were summed, and aggregated by age and SES. The measure of SES used was the Scottish Index of Multiple Deprivation (SIMD) [24]. This is a small-area-based ranked measure which combines 38 indicators of deprivation across seven different conceptual domains, and is recognised by the Scottish Government as the standard method of assessing deprivation in Scotland. For the purposes of this investigation, rankings of the 6505 geographical areas recorded in the SIMD were expressed as quintiles (the first and fifth quintiles representing the most- and least-deprived groups in the population, respectively). Numbers of person-years and of deaths accrued by the type 2 diabetes cohort were subtracted from mid-year population totals and overall numbers of deaths in Scotland, these national data again being supplied by ISD and aggregated by age and SES (quintile of the SIMD). This subtraction operation generated the comparison group (referred to hereafter as the ‘type 2 diabetes-free population’), from which person-years and deaths contributed by people with type 2 diabetes had been removed. This approach avoids the inadvertent introduction of bias from selection of a limited number of controls. Because the study was conceived as a strict comparison between groups with and without type 2 diabetes, the type 2 diabetes-free population retained a small number of individuals with type 1 diabetes. However, the effect of these people on life expectancy in the type 2 diabetes-free population is expected to be small, because type 1 disease is uncommon (relative to total population size) in the age range covered by the study (40–89 years).

For both the type 2 diabetes cohort and the type 2 diabetes-free population, abridged life tables as described by Chiang [25] were constructed to generate life-expectancy estimates. Creation of the life tables was achieved by adapting an Excel template published by the UK Office of National Statistics [26]. Operation of the template was verified using an S-language routine for generating life tables published by Selvin [27]; both approaches yielded identical results. Because type 2 diabetes is rare in younger adults, and elderly people are subject to a wide range of mortality risks beyond those specifically associated with diabetes, life-expectancy values were reported only for the age range 40–89 years (in five-year intervals: 40–44, 45–49, 50–54... ...80–84, 85–89). Five-year intervals are conventionally chosen for life-table approaches because of the exponential, rapidly changing effect of increasing age on life expectancy. Stratification by age is used to investigate the presence or absence of interactions, in this case with both diabetes status and SES. Unlike regression-model-based analyses, the life-table approach does not allow formal tests for interaction (which can also be difficult to interpret when there are multiple interactions as in this case between type 2 diabetes, age, sex and SES). We have used figures to illustrate how SES affects the association between type 2 diabetes and life expectancy. Results were derived separately for each sex, yielding a total of 100 paired life-expectancy values (two sexes × five SES strata × ten age intervals). Each pair of values consisted of two elements: the estimated life expectancy for the type 2 diabetes cohort, and the corresponding value for the type 2 diabetes-free population. Paired values were also expressed as the difference between the estimated life expectancy for the type 2 diabetes cohort and that for the type 2 diabetes-free population, calculated as the former minus the latter. Thus, negative difference values indicated a lower expectation of life in people with type 2 diabetes. CIs around life-expectancy values, and around differences in life-expectancy values, were calculated using the methods described by Chiang [25]. Although the study period was defined as 2012–2014, all analyses were repeated for the period 2004–2006 with the aim of informally assessing whether the main findings of interest were materially different for this earlier time interval.

## Results

Results were based on 272,597 individuals with type 2 diabetes and approximately 2.75 million people without type 2 diabetes, the latter total being that for the year 2013 (the middle calendar year of the study period). Over the three-year study period, numbers of person-years accrued for the type 2 diabetes cohort in the age range of interest (40–89 years) were 390,567 (men) and 306,872 (women). Corresponding numbers of deaths were 13,875 (men) and 11,471 (women). For the type 2 diabetes-free population, numbers of person-years in the target age range were 3,509,683 (men) and 4,034,066 (women). Numbers of deaths in the type 2 diabetes-free population were 53,711 (men) and 53,705 (women). Table 1 summarises results without stratification by SES in order to illustrate the overall effects of type 2 diabetes on life expectancy.

**Table 1:** Abridged life table for men and women with type 2 diabetes vs population of Scotland without type 2 diabetes; period 2012–2014

	Observed data in type 2 diabetes cohort			Observed data in type 2 diabetes-free population			Estimated life expectancy (95% CI)		
Age interval (years)	Person-years	Deaths	Death rate per 1000 P-Y	Person-years	Deaths	Death rate per 1000 P-Y	Type 2 diabetes cohort	Type 2 diabetes-free population	LE difference^a^
Men									
40–44	12,858	64	5.0	529,939	1305	2.5	35.3 (35.0, 35.7)	39.4 (39.3, 39.4)	−4.1 (−4.4, −3.8)
45–49	23,795	125	5.3	569,618	1811	3.2	31.2 (30.9, 31.4)	34.8 (34.7, 34.9)	−3.6 (−3.8, −3.4)
50–54	36,860	300	8.1	537,661	2368	4.4	26.9 (26.7, 27.1)	30.3 (30.3, 30.4)	−3.4 (−3.6, −3.2)
55–59	48,318	559	11.6	460,249	3200	7.0	23.0 (22.8, 23.1)	26.0 (25.9, 26.0)	−3.0 (−3.2, −2.8)
60–64	58,216	990	17.0	408,713	4348	10.6	19.2 (19.0, 19.3)	21.8 (21.7, 21.9)	−2.6 (−2.8, −2.4)
65–69	65,217	1546	23.7	363,325	6118	16.8	15.7 (15.5, 15.8)	17.8 (17.8, 17.9)	−2.1 (−2.3, −1.9)
70–74	55,740	2246	40.3	255,444	7160	28.0	12.3 (12.2, 12.4)	14.2 (14.1, 14.2)	−1.9 (−2.0, −1.8)
75–79	47,135	2991	63.5	193,469	8779	45.4	9.5 (9.4, 9.6)	10.9 (10.9, 11.0)	−1.4 (−1.5, −1.3)
80–84	29,732	2978	100.2	127,063	9812	77.2	7.2 (7.0, 7.3)	8.1 (8.0, 8.2)	−0.9 (−1.0, −0.8)
85–89	12,696	2076	163.5	64,202	8810	137.2	5.3 (5.2, 5.4)	5.8 (5.7, 5.8)	−0.5 (−0.6, −0.4)
Women									
40–44	9116	30	3.3	568,674	776	1.4	37.2 (36.8, 37.5)	42.6 (42.6, 42.7)	−5.4 (−5.8, −5.0)
45–49	15,936	73	4.6	612,448	1190	1.9	32.7 (32.5, 33.0)	37.9 (37.9, 38.0)	−5.2 (−5.5, −4.9)
50–54	23,908	174	7.3	577,364	1751	3.0	28.4 (28.2, 28.7)	33.3 (33.2, 33.3)	−4.9 (−5.2, −4.6)
55–59	31,453	302	9.6	500,295	2322	4.6	24.4 (24.2, 24.6)	28.7 (28.7, 28.8)	−4.3 (−4.5, −4.1)
60–64	38,141	530	13.9	450,705	3202	7.1	20.5 (20.3, 20.7)	24.4 (24.3, 24.4)	−3.9 (−4.1, −3.7)
65–69	44,864	999	22.3	415,240	4529	10.9	16.8 (16.6, 16.9)	20.1 (20.1, 20.2)	−3.3 (−3.5, −3.1)
70–74	46,212	1523	33.0	318,672	6036	18.9	13.5 (13.3, 13.6)	16.1 (16.1, 16.2)	−2.6 (−2.7, −2.5)
75–79	45,181	2433	53.8	264,813	8592	32.4	10.4 (10.3, 10.5)	12.5 (12.4, 12.5)	−2.1 (−2.2, −2.0)
80–84	33,888	2861	84.4	200,719	11,853	59.1	7.9 (7.8, 8.0)	9.3 (9.2, 9.3)	−1.4 (−1.5, −1.3)
85–89	18,174	2546	140.1	125,136	13,454	107.5	5.8 (5.7, 5.9)	6.6 (6.6, 6.6)	−0.8 (−0.9, −0.7)

^a^Difference calculated as: type 2 diabetes cohort life expectancy minus type 2 diabetes-free population life expectancy; negative values indicate lower life expectancy in type 2 diabetes cohort

LE, life expectancy; P-Y, person-years

**Fig. 1:**
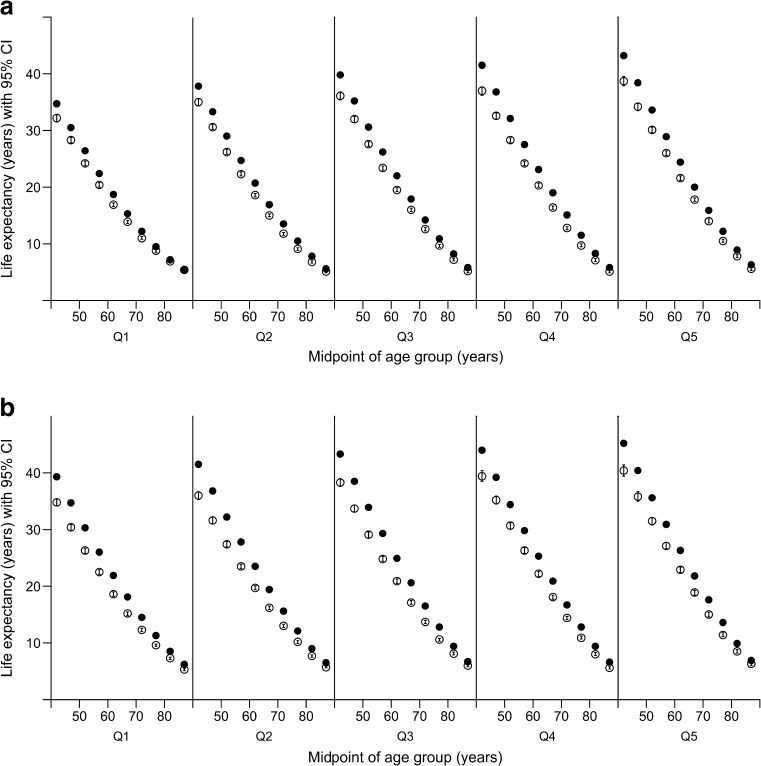
Life expectancy in men (**a**) and women (**b**) for the type 2 diabetes cohort (white symbols) and type 2 diabetes-free population (black symbols) in Scotland, period 2012–2014, stratified by SIMD quintile (Q1 = most deprived). The horizontal axis shows the midpoint of age interval (e.g. age interval 40–45 is plotted at 42). The vertical axis shows life expectancy (years). Error bars represent 95% CI

Table 1 presents, for each combination of sex and age interval, the numbers of person-years and of deaths in both cohorts; observed mortality rates; estimated life-expectancy values (with 95% CI); and the difference in life expectancy between the type 2 diabetes cohort and the type 2 diabetes-free population (with 95% CI). The results show that type 2 diabetes is associated with lower life expectancy at all ages and that difference in life expectancy between people with and without type 2 diabetes is greater at younger than older ages and for women than men. Values of life expectancy for both groups (with stratification by SES) are shown graphically in Fig. 1a and 1b for men and women respectively with data given in ESM Tables 1–5 in the supplementary material.

**Fig. 2:**
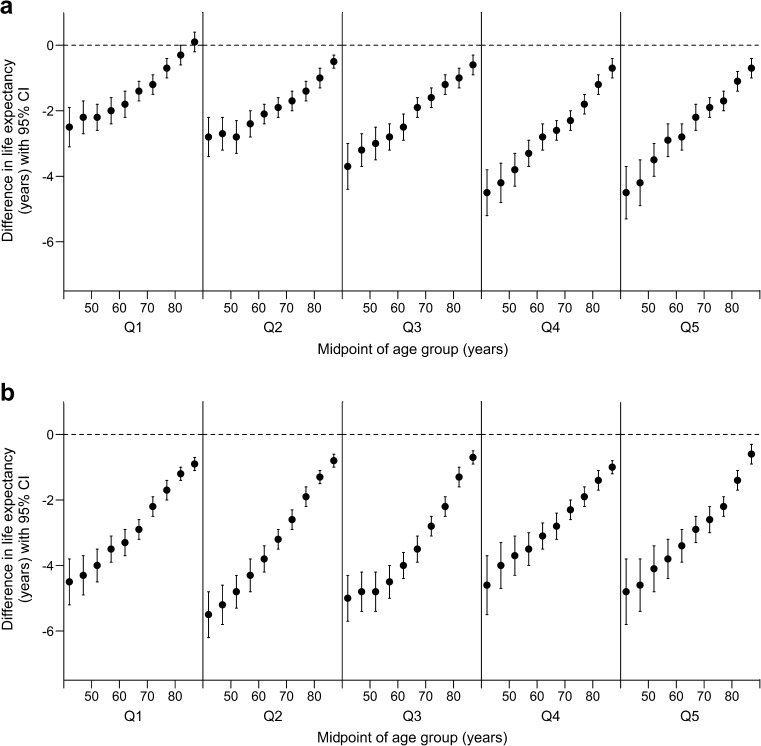
Difference in life expectancy in men (**a**) and women (**b**) in Scotland, period 2012–2014; data are calculated as the value for type 2 diabetes cohort minus value for type 2 diabetes-free population; negative values indicate lower life expectancy in the type 2 diabetes cohort. Data are stratified by SIDM quintile (Q1 = most deprived). The horizontal axis shows the midpoint of age interval (e.g. age interval 40–45 is plotted at 42). The vertical axis shows the difference in life expectancy (years); the dashed horizontal line marks zero (‘no difference’). Error bars represent 95% CI

Differences in life expectancy between those with type 2 diabetes and the corresponding stratum in the type 2 diabetes-free population are presented in Fig. 2a and b for men and women respectively. In addition to the patterns by age demonstrated in Table 1, these figures demonstrate that SES also influences life expectancy, with the smallest differences in life expectancy between men with and without diabetes observed in the most-deprived fifth of the population (identified as Q1 in the figures).

Differences in life expectancy (negative values indicating reduced expectancy of life in those with type 2 diabetes) ranged from 0.1 years (−0.2 years to 0.4 years) for men aged 85–89 in the most-deprived quintile of SIMD to −5.5 years (−6.2 years to −4.8 years) for women aged 40–44 in the second most-deprived quintile of SIMD. There were only two instances among the 100 calculated life-expectancy differences for which the 95% CI was found to include the value zero (interpreted as insufficient evidence to conclude that a statistically significant difference in life expectancy exists between those with type 2 diabetes and those without), and these occurred among the most-deprived fifth of men in the 80–84 and 85–89 year age groups. For all remaining life-expectancy value pairs, the 95% CI around the difference excluded zero: the intervals were strictly negative, indicating statistically significant differences.

**Fig. 3:**
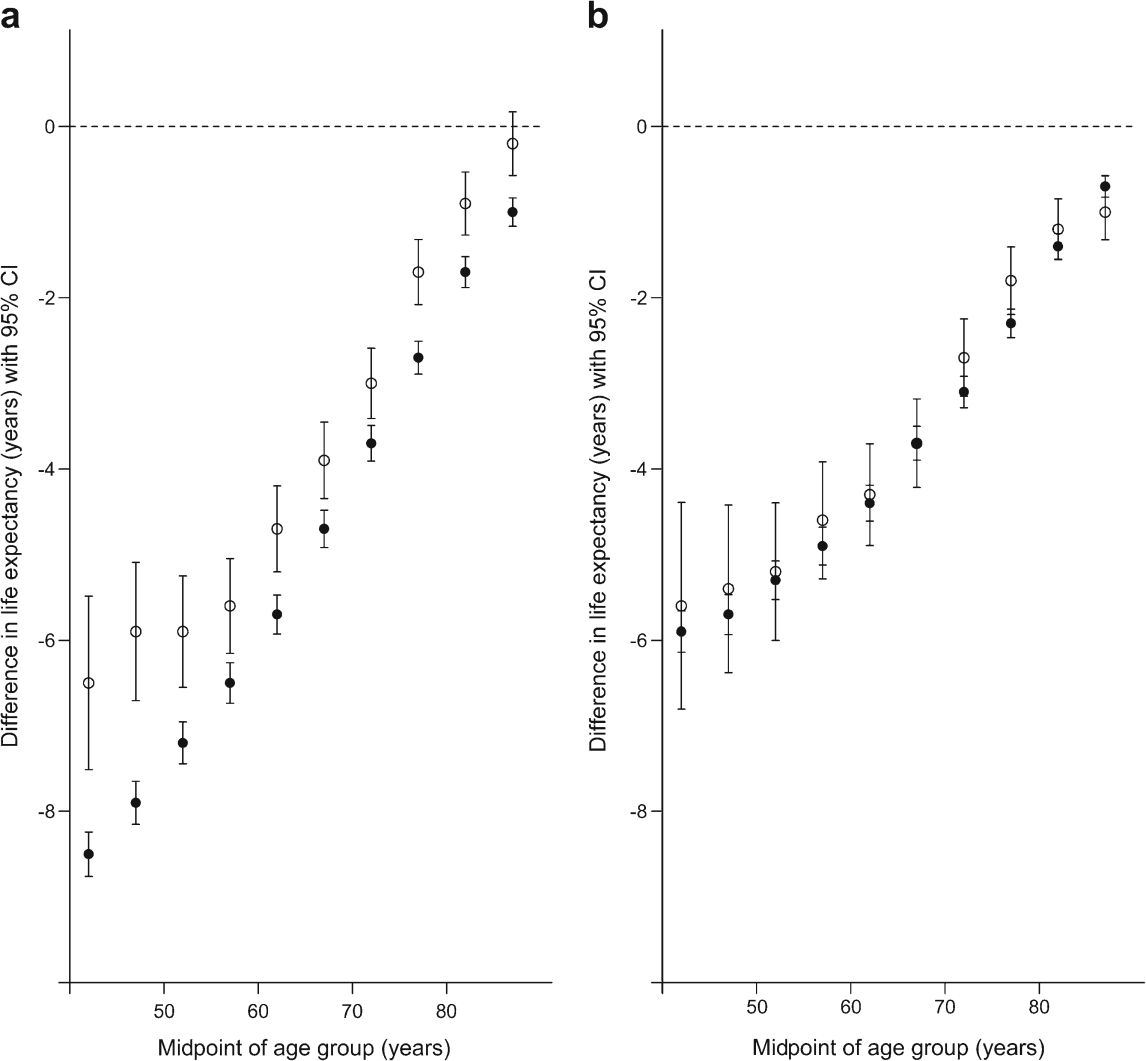
Difference in life expectancy in men (**a**) and women (**b**) in Scotland, period 2012–2014; data are calculated as the value for most-deprived minus value for least-deprived SIMD quintile; negative values indicate lower life expectancy in the most-deprived quintile. White symbols, type 2 diabetes cohort; black symbols, type 2 diabetes-free population. The horizontal axis shows the midpoint of age interval (e.g. age interval 40–45 is plotted at 42). The vertical axis shows the difference in life expectancy (years); the dashed horizontal line marks zero (‘no difference’). Error bars represent 95% CI

The study’s second objective—assessing whether SES-related differences in life expectancy in the type 2 diabetes cohort were similar to those in the type 2 diabetes-free population—was pursued by calculating the difference between the respective life-expectancy values for people in the most- and least-deprived SIMD quintiles for each combination of cohort, sex and age interval (40 strata in total). This quantity may be viewed as an approximation to the gradient of SES-related differences in life expectancy (assuming a broad linearity in the association between life expectancy and SES within each stratum). These differences are presented in Fig. 3a and b for men and women respectively, which show that life expectancy is lower for people in the most- compared with the least-deprived fifths of the population for almost all age groups regardless of type 2 diabetes status, with the exception of men of 85–89 years of age with type 2 diabetes. The difference in life expectancy between people in the most- and least-deprived fifths of the population is greater at younger than older ages and for men without type 2 diabetes than for men with type 2 diabetes; the effect of deprivation on life expectancy was similar in women with and without type 2 diabetes.

## Discussion

This is believed to be the first study investigating the life-expectancy deficit associated with type 2 diabetes in a complete national population to focus specifically on the potential role of SES (an accepted determinant of mortality risk in both diabetic and general populations). The study’s first objective was to ascertain whether differences in life expectancy associated with type 2 diabetes are present at all levels of SES and to quantify any such differences. The findings indicate that, with the exception of deprived elderly men (most-deprived SIMD quintile; ages 80–84 and 85–89), life expectancy in those with type 2 diabetes is significantly lower than that in the type 2 diabetes-free population at all ages and levels of SES. While persistence of a life expectancy deficit across the full range of deprivation levels is confirmed, it is clear that the magnitude of the differences in life expectancy between those with and without type 2 diabetes is not uniform. A marked distinction by sex is evident: type 2 diabetes-related differences in life expectancy are generally lower in absolute terms (i.e. closer to zero) in men than in women, especially at the more deprived end of the SES spectrum (Fig. 2a and Fig. 2b). The study’s second objective was to assess whether the patterns of SES-related differences in life expectancy among people with type 2 diabetes are similar to those evident in a general population. For men, Fig. 3a suggests that the difference in life expectancy between the most- and least-deprived groups with type 2 diabetes is consistently significantly lower (in absolute terms) for all age intervals than that in the corresponding stratum of the type 2 diabetes-free population. That is, the SES-related difference in mortality risk observed in the ‘general’ male population appears to be attenuated in those with type 2 diabetes. For women, (Fig. 3b) the position is different: in most age intervals, the SES-related contrast in life expectancy for those with type 2 diabetes is not significantly different from that observed in the type 2 diabetes-free population (as indicated by overlapping CIs).

Two strengths of the study may be highlighted. First, the use of whole-population data avoids many of the biases which may potentially arise in studies based on limited samples, for example, people resident in a specific, relatively small geographical area. An example of the latter is provided by the previously cited investigation reported by Perna et al [17]. This study, which focused on socioeconomic differences in life expectancy among people with diabetes, used data for the Augsburg region of Bavaria (Germany). The authors of this study describe their cohort as a sample ‘whose population structure reflects that of Germany as a whole’ (Perna et al [17] p. 2), but this is obviously no substitute for having access (as in the study reported here) to whole-population data. Indeed, Perna et al openly acknowledge in their Discussion biases attributable to the composition of their sample. A second strength of the present study relates to the definition of diabetes status. While some studies in the area of diabetes and mortality have relied on self-reporting of the disease, the SCI-Diabetes registry provides a reasonably robust, clinically confirmed representation of diabetes status.

Against these strengths, limitations of the investigation reported here are acknowledged. Using an area-based measure of SES does not reliably attribute SES to individuals. However, SIMD is based on a small average population of about 800 people. SIMD is believed to portray individual SES adequately for health-related research in Scotland, and area-based measures are increasingly used internationally to assess inequalities in health and mortality [28]. A second limitation relates to the fundamental nature of life tables. The life table as employed here estimates the period life expectancy, defined by the UK Office for National Statistics as ‘the average number of years a person would live, if he or she experienced the particular area’s age-specific mortality rates for that time period throughout his or her life’ [29]. While this measure is commonly used to compare life expectancy between groups or countries or over time, the assumption of static mortality rates is obviously unrealistic. The alternative is to estimate cohort life expectancy, which essentially either uses data for a cohort that has been followed up to complete mortality or else focuses on synthetic cohorts wherein assumptions are made about the future patterns in mortality rates and differentials. Such an approach has the advantage of avoiding the simplistic assumption that life expectancy will not change in future but has the drawback that it is very susceptible to arbitrary assumptions about future rates. A third limitation relates to the possible misclassification of type of diabetes; specifically, the potential for people with insulin-treated type 2 diabetes to be labelled as having type 1 disease. Where this occurs, these individuals (who have a relatively high risk of mortality) will be included in the type 2 diabetes-free population and thus obscure the ‘true’ contrast between those with and without type 2 diabetes. While such misclassification cannot be ruled out, the algorithm used in SCI-Diabetes to define type of diabetes has been extensively refined and is believed to be robust. Consequently, instances of misclassification are likely to be rare.

Two aspects of the study’s findings merit further comment. First, although a life-expectancy deficit associated with type 2 diabetes is observed at all ages, its absolute magnitude (the number of expected years ‘lost’) is relatively small, especially at older ages during which type 2 diabetes becomes more common. A meta-analysis cited earlier identified an approximate doubling of mortality risk in people with diabetes [3], but when viewed from the alternative age-specific perspective provided by life expectancy, the increased ‘vulnerability to death’ associated with type 2 diabetes is perhaps smaller than might intuitively have been expected. A second prominent feature of the results is that patterns of the absolute differences in life expectancy associated with type 2 diabetes, and the respective sizes of the differences associated with type 2 diabetes and with inequality (SES), vary markedly by sex. In men, the difference in life expectancy associated with type 2 diabetes (Fig. 2a) is smaller than that associated with deprivation (more specifically, the contrast between the most- and least-deprived population quintiles, Fig. 3a). This feature is largely absent in women. Given the pervasiveness of socially patterned mortality found in innumerable societies and contexts, the substantial association of SES with life expectancy in men is unsurprising, and reinforces the message that for men with diabetes (as for many other clinical entities), improvement of mortality prospects will demand not only effective management of the condition but also the development of more general strategies to reduce inequality. However, reasons for the between-sex difference require consideration. A major contributor to premature death in people with diabetes is CHD, and previous work suggests that diabetes is a more influential risk factor for coronary death in women than in men [30]. However, Kanaya et al reported that the elevation of CHD mortality in women with diabetes relative to that in men with the disease is mediated by established cardiovascular disease risk factors, and largely disappears after adjustment for these factors [31]. It may therefore be that sex-related differences in the associations between diabetes and life expectancy (notably, the respective effect sizes associated with type 2 diabetes and with SES) are wholly or partly explained by differential patterns of established cardiovascular disease risk factors or comorbidity (with potential variation by sex and/or SES and/or diabetes status). We were not able to test this hypothesis because risk factor data are not available for people without diabetes in this study.

Scope exists for further work to expand understanding of the relationships that link diabetes, SES, and life expectancy, including the role of differing patterns of cardiovascular disease risk factors and comorbidities. The study considered only the presence or absence of type 2 diabetes, but took no account of the possible effect of duration of diabetes on the observed associations. For example, a person aged 55 might have recently been diagnosed with the disease, or alternatively might have lived with the condition for decades. Previous work has demonstrated that duration of diabetes appears to modify the association between type 2 diabetes and mortality [4]. It is therefore reasonable to hypothesise that the reduction in life expectancy for those with type 2 diabetes observed in this study may be underestimated for individuals with long-standing diabetes, and overestimated for those recently diagnosed with the condition. Further work is required to clarify this. A related question, again unanswered in this investigation, is whether age at onset of diabetes modifies the relationship of the disease with life expectancy. The results presented here suggest that for older people the absolute reduction in life expectancy associated with type 2 diabetes is small, but introducing age at onset into consideration would reveal whether onset of diabetes at more advanced ages exerts any material effect (in a statistical, not causal, sense) on life expectancy at all.

In conclusion, we have shown that type 2 diabetes is associated with reductions in life expectancy in most subgroups of the population of Scotland and that the size of the reduction varies by age, sex and SES. Even the largest differences in life expectancy associated with type 2 diabetes are smaller than may have been expected from relative risks of mortality associated with type 2 diabetes that we and others have previously reported. However, these findings suggest that increasing prevalence of diabetes will contribute to slower improvements in population life expectancy over time. Increasing incidence of type 2 diabetes in young people can be expected to have a particularly detrimental effect on life expectancy.

## Electronic supplementary material

ESM Tables (PDF 237 kb)

## Abbreviations

ISD: Information Services Division (organisational unit of UK National Health Service in Scotland)
NHSNSS: National Health Service National Services Scotland (organisational unit of UK National Health Service in Scotland, charged with provision of common services to individual health boards)
SCI-Diabetes: Scottish Care Information – Diabetes (national register of Scottish patients with diabetes)
SES: Socioeconomic status
SIMD: Scottish Index of Multiple Deprivation
