# Supplementary material for: Type 2 diabetes, socioeconomic status and life expectancy in Scotland (2012–2014): a population-based observational study
Source: Diabetologia. 2017 Oct 26;61(1):108–16. doi: 10.1007/s00125-017-4478-x (PMC6448945; doi:10.1007/s00125-017-4478-x)
Supplement: Supplementary file 1 — (PDF 237 kb) [file 125_2017_4478_MOESM1_ESM.pdf]

ESM Table 1

Abridged life table for men and women with type 2 diabetes vs. population of Scotland without type 2 diabetes; SIMD quintile 1 (most deprived), period 2012-2014

	observed data in type 2 diabetes cohort			observed data in type 2 diabetes-free population			estimated life expectancy (95% confidence interval)		
age interval (years)	person-years	deaths	death rate per 1,000 p-y	person-years	deaths	death rate per 1,000 p-y	type 2 diabetes cohort	type 2 diabetes-free population	LE difference[a]
MEN									
40-44	3,863.3	29	7.5	101,217.7	523	5.2	32.2 (31.5, 32.8)	34.7 (34.5, 34.8)	-2.5 (-3.1, -1.9)
45-49	6,674.1	46	6.9	106,663.9	669	6.3	28.3 (27.8, 28.8)	30.5 (30.3, 30.7)	-2.2 (-2.7, -1.7)
50-54	9,888.4	107	10.8	97,830.6	825	8.4	24.2 (23.8, 24.6)	26.4 (26.2, 26.6)	-2.2 (-2.6, -1.8)
55-59	11,928.5	190	15.9	80,310.5	1,002	12.5	20.4 (20.0, 20.8)	22.4 (22.2, 22.6)	-2.0 (-2.4, -1.6)
60-64	12,844.0	329	25.6	68,876.0	1,304	18.9	16.9 (16.6, 17.2)	18.7 (18.5, 18.9)	-1.8 (-2.2, -1.4)
65-69	13,429.2	473	35.2	58,620.8	1,625	27.7	13.9 (13.6, 14.1)	15.3 (15.2, 15.5)	-1.4 (-1.7, -1.1)
70-74	11,648.9	643	55.2	43,153.1	1,853	42.9	11.0 (10.8, 11.3)	12.2 (12.1, 12.4)	-1.2 (-1.5, -0.9)
75-79	9,511.6	756	79.5	32,407.4	2,085	64.3	8.8 (8.5, 9.0)	9.5 (9.4, 9.7)	-0.7 (-1.0, -0.4)
80-84	5,488.8	629	114.6	21,123.2	2,051	97.1	6.9 (6.6, 7.2)	7.2 (7.1, 7.4)	-0.3 (-0.6, -0.0)
85-89	2,166.1	353	163.0	10,073.9	1,685	167.3	5.4 (5.2, 5.7)	5.3 (5.2, 5.4)	0.1 (-0.2, 0.4)
WOMEN									
40-44	3,037.9	11	3.6	111,048.1	272	2.4	34.8 (34.2, 35.4)	39.3 (39.1, 39.5)	-4.5 (-5.2, -3.8)
45-49	5,153.0	32	6.2	116,164.0	379	3.3	30.4 (29.8, 30.9)	34.7 (34.6, 34.9)	-4.3 (-4.9, -3.7)
50-54	7,171.1	72	10.0	106,848.9	518	4.8	26.3 (25.8, 26.7)	30.3 (30.1, 30.4)	-4.0 (-4.5, -3.5)
55-59	8,862.6	96	10.8	89,301.4	696	7.8	22.5 (22.1, 22.9)	26.0 (25.8, 26.1)	-3.5 (-3.9, -3.1)
60-64	9,851.9	182	18.5	75,593.1	887	11.7	18.6 (18.2, 19.0)	21.9 (21.7, 22.0)	-3.3 (-3.7, -2.9)
65-69	10,810.7	338	31.3	67,723.3	1,188	17.5	15.2 (14.8, 15.5)	18.1 (17.9, 18.2)	-2.9 (-3.2, -2.6)
70-74	11,356.3	473	41.7	56,889.7	1,564	27.5	12.3 (12.0, 12.6)	14.5 (14.4, 14.6)	-2.2 (-2.5, -1.9)
75-79	10,956.7	710	64.8	49,068.3	2,240	45.7	9.6 (9.4, 9.8)	11.3 (11.1, 11.4)	-1.7 (-2.0, -1.4)
80-84	7,686.6	732	95.2	37,434.4	2,744	73.3	7.3 (7.1, 7.5)	8.5 (8.4, 8.6)	-1.2 (-1.4, -1.0)
85-89	3,661.7	570	155.7	21,921.3	2,734	124.7	5.3 (5.1, 5.5)	6.2 (6.1, 6.3)	-0.9 (-1.1, -0.7)

[a] Difference calculated as (type 2 diabetes cohort life expectancy minus type 2 diabetes-free population life expectancy); negative values indicate lower life expectancy in type 2 diabetes cohort.

ESM Table 2

Abridged life table for men and women with type 2 diabetes vs. population of Scotland without type 2 diabetes; SIMD quintile 2, period 2012-2014

	observed data in type 2 diabetes cohort			observed data in type 2 diabetes-free population			estimated life expectancy (95% confidence interval)		
age interval (years)	person-years	deaths	death rate per 1,000 p-y	person-years	deaths	death rate per 1,000 p-y	type 2 diabetes cohort	type 2 diabetes-free population	LE difference[a]
MEN									
40-44	3,123.0	12	3.8	102,707.0	312	3.0	35.0 (34.4, 35.6)	37.8 (37.6, 38.0)	-2.8 (-3.4, -2.2)
45-49	5,720.1	24	4.2	107,585.9	441	4.1	30.6 (30.2, 31.1)	33.3 (33.2, 33.5)	-2.7 (-3.2, -2.2)
50-54	8,535.9	77	9.0	101,610.1	534	5.3	26.2 (25.8, 26.7)	29.0 (28.8, 29.1)	-2.8 (-3.3, -2.3)
55-59	10,737.6	136	12.7	86,929.4	777	8.9	22.3 (22.0, 22.7)	24.7 (24.5, 24.8)	-2.4 (-2.8, -2.0)
60-64	12,829.1	213	16.6	77,059.0	989	12.8	18.6 (18.3, 18.9)	20.7 (20.5, 20.8)	-2.1 (-2.4, -1.8)
65-69	14,159.6	364	25.7	69,092.4	1,430	20.7	15.0 (14.8, 15.3)	16.9 (16.7, 17.0)	-1.9 (-2.2, -1.6)
70-74	12,414.7	554	44.6	50,090.3	1,671	33.4	11.8 (11.5, 12.0)	13.5 (13.3, 13.6)	-1.7 (-2.0, -1.4)
75-79	10,279.6	704	68.5	38,548.4	1,962	50.9	9.1 (8.8, 9.3)	10.5 (10.3, 10.6)	-1.4 (-1.7, -1.1)
80-84	6,306.9	706	111.9	25,390.2	2,135	84.1	6.8 (6.6, 7.0)	7.8 (7.7, 7.9)	-1.0 (-1.3, -0.7)
85-89	2,682.1	443	165.2	12,937.9	1,882	145.5	5.1 (4.9, 5.4)	5.6 (5.5, 5.7)	-0.5 (-0.7, -0.3)
WOMEN									
40-44	2,321.0	8	3.4	109,434.0	196	1.8	36.0 (35.3, 36.7)	41.5 (41.3, 41.6)	-5.5 (-6.2, -4.8)
45-49	3,729.8	21	5.6	116,186.2	280	2.4	31.6 (31.0, 32.2)	36.8 (36.7, 37.0)	-5.2 (-5.8, -4.6)
50-54	5,621.4	48	8.5	110,818.6	396	3.6	27.4 (26.9, 27.9)	32.2 (32.1, 32.4)	-4.8 (-5.3, -4.3)
55-59	7,468.5	85	11.4	95,488.5	536	5.6	23.5 (23.1, 23.9)	27.8 (27.6, 27.9)	-4.3 (-4.8, -3.8)
60-64	9,029.3	148	16.4	86,690.7	731	8.4	19.7 (19.4, 20.1)	23.5 (23.3, 23.6)	-3.8 (-4.2, -3.4)
65-69	10,875.2	269	24.7	80,618.8	1,079	13.4	16.2 (15.9, 16.5)	19.4 (19.3, 19.5)	-3.2 (-3.5, -2.9)
70-74	11,048.8	415	37.6	64,305.2	1,448	22.5	13.0 (12.7, 13.3)	15.6 (15.4, 15.7)	-2.6 (-2.9, -2.3)
75-79	10,758.2	614	57.1	56,316.8	2,046	36.3	10.2 (9.9, 10.4)	12.1 (12.0, 12.2)	-1.9 (-2.2, -1.6)
80-84	7,943.3	711	89.5	42,955.7	2,743	63.9	7.7 (7.5, 7.9)	9.0 (8.9, 9.1)	-1.3 (-1.5, -1.1)
85-89	4,153.3	572	137.7	26,697.7	3,023	113.2	5.7 (5.6, 5.9)	6.5 (6.4, 6.6)	-0.8 (-1.0, -0.6)

[a] Difference calculated as (type 2 diabetes cohort life expectancy minus type 2 diabetes-free population life expectancy); negative values indicate lower life expectancy in type 2 diabetes cohort.

ESM Table 3

Abridged life table for men and women with type 2 diabetes vs. population of Scotland without type 2 diabetes; SIMD quintile 3, period 2012-2014

	observed data in type 2 diabetes cohort			observed data in type 2 diabetes-free population			estimated life expectancy (95% confidence interval)		
age interval (years)	person-years	deaths	death rate per 1,000 p-y	person-years	deaths	death rate per 1,000 p-y	type 2 diabetes cohort	type 2 diabetes-free population	LE difference[a]
MEN									
40-44	2,455.8	12	4.9	107,576.2	234	2.2	36.1 (35.4, 36.8)	39.8 (39.6, 39.9)	-3.7 (-4.4, -3.0)
45-49	4,601.7	20	4.3	116,446.3	286	2.5	32.0 (31.4, 32.5)	35.2 (35.0, 35.3)	-3.2 (-3.7, -2.7)
50-54	7,169.5	42	5.9	109,923.6	485	4.4	27.6 (27.2, 28.0)	30.6 (30.4, 30.7)	-3.0 (-3.5, -2.5)
55-59	9,505.2	104	10.9	94,466.8	590	6.2	23.4 (23.0, 23.7)	26.2 (26.0, 26.4)	-2.8 (-3.2, -2.4)
60-64	11,954.4	204	17.1	85,436.6	836	9.8	19.5 (19.2, 19.9)	22.0 (21.8, 22.1)	-2.5 (-2.9, -2.1)
65-69	13,733.6	296	21.6	77,613.4	1,253	16.1	16.0 (15.7, 16.3)	17.9 (17.8, 18.1)	-1.9 (-2.2, -1.6)
70-74	11,827.3	446	37.7	54,955.7	1,492	27.1	12.6 (12.3, 12.9)	14.2 (14.1, 14.4)	-1.6 (-1.9, -1.3)
75-79	10,014.0	600	59.9	41,093.0	1,911	46.5	9.7 (9.4, 9.9)	10.9 (10.8, 11.1)	-1.2 (-1.5, -0.9)
80-84	6,346.9	603	95.0	26,556.1	2,028	76.4	7.2 (7.0, 7.4)	8.2 (8.0, 8.3)	-1.0 (-1.3, -0.7)
85-89	2,639.7	440	166.7	13,567.3	1,777	131.0	5.2 (4.9, 5.4)	5.8 (5.7, 5.9)	-0.6 (-0.9, -0.3)
WOMEN									
40-44	1,705.4	4	2.3	113,476.6	127	1.1	38.3 (37.6, 39.0)	43.3 (43.1, 43.5)	-5.0 (-5.7, -4.3)
45-49	3,098.3	7	2.3	122,792.7	226	1.8	33.7 (33.1, 34.3)	38.5 (38.4, 38.7)	-4.8 (-5.4, -4.2)
50-54	4,743.7	27	5.7	116,178.3	322	2.8	29.1 (28.5, 29.6)	33.9 (33.7, 34.0)	-4.8 (-5.4, -4.2)
55-59	6,099.5	59	9.7	102,061.5	408	4.0	24.8 (24.4, 25.3)	29.3 (29.2, 29.5)	-4.5 (-5.0, -4.0)
60-64	7,544.7	94	12.5	93,757.3	607	6.5	20.9 (20.5, 21.3)	24.9 (24.7, 25.0)	-4.0 (-4.4, -3.6)
65-69	8,971.1	180	20.1	87,956.9	855	9.7	17.1 (16.8, 17.5)	20.6 (20.5, 20.7)	-3.5 (-3.9, -3.1)
70-74	9,457.6	298	31.5	67,230.4	1,177	17.5	13.7 (13.4, 14.0)	16.5 (16.4, 16.6)	-2.8 (-3.1, -2.5)
75-79	8,867.6	486	54.8	53,878.4	1,602	29.7	10.6 (10.3, 10.8)	12.8 (12.7, 12.9)	-2.2 (-2.5, -1.9)
80-84	6,676.3	538	80.6	40,816.7	2,356	57.7	8.1 (7.9, 8.4)	9.4 (9.3, 9.5)	-1.3 (-1.6, -1.0)
85-89	3,797.8	504	132.7	25,738.2	2,670	103.7	6.0 (5.8, 6.2)	6.7 (6.7, 6.8)	-0.7 (-0.9, -0.5)

[a] Difference calculated as (type 2 diabetes cohort life expectancy minus type 2 diabetes-free population life expectancy); negative values indicate lower life expectancy in type 2 diabetes cohort.

ESM Table 4

Abridged life table for men and women with type 2 diabetes vs. population of Scotland without type 2 diabetes; SIMD quintile 4, period 2012-2014

	observed data in type 2 diabetes cohort			observed data in type 2 diabetes-free population			estimated life expectancy (95% confidence interval)		
age interval (years)	person-years	deaths	death rate per 1,000 p-y	person-years	deaths	death rate per 1,000 p-y	type 2 diabetes cohort	type 2 diabetes-free population	LE difference[a]
MEN									
40-44	1,962.1	7	3.6	111,406.9	139	1.2	37.0 (36.2, 37.7)	41.5 (41.4, 41.7)	-4.5 (-5.2, -3.8)
45-49	3,857.0	18	4.7	121,384.0	248	2.0	32.6 (32.0, 33.1)	36.8 (36.6, 36.9)	-4.2 (-4.8, -3.6)
50-54	6,183.7	42	6.8	115,246.3	316	2.7	28.3 (27.8, 28.7)	32.1 (32.0, 32.3)	-3.8 (-4.3, -3.3)
55-59	8,739.7	90	10.3	100,259.3	490	4.9	24.2 (23.8, 24.6)	27.5 (27.4, 27.7)	-3.3 (-3.7, -2.9)
60-64	11,206.9	132	11.8	90,043.1	696	7.7	20.3 (20.0, 20.7)	23.1 (23.0, 23.3)	-2.8 (-3.2, -2.4)
65-69	13,034.7	244	18.7	80,299.3	1,032	12.9	16.4 (16.1, 16.7)	19.0 (18.8, 19.1)	-2.6 (-2.9, -2.3)
70-74	10,935.6	384	35.1	55,600.4	1,209	21.7	12.8 (12.5, 13.0)	15.1 (14.9, 15.2)	-2.3 (-2.6, -2.0)
75-79	9,337.0	532	57.0	41,055.0	1,523	37.1	9.7 (9.5, 10.0)	11.5 (11.4, 11.6)	-1.8 (-2.1, -1.5)
80-84	6,224.0	604	97.0	27,202.0	1,906	70.1	7.1 (6.9, 7.4)	8.3 (8.2, 8.5)	-1.2 (-1.5, -0.9)
85-89	2,696.3	464	172.1	13,567.7	1,833	135.1	5.1 (4.9, 5.3)	5.8 (5.7, 5.9)	-0.7 (-1.0, -0.4)
WOMEN									
40-44	1,227.4	5	4.1	119,229.6	110	0.9	39.4 (38.5, 40.4)	44.0 (43.8, 44.2)	-4.6 (-5.5, -3.7)
45-49	2,362.0	7	3.0	129,753.0	168	1.3	35.2 (34.5, 35.9)	39.2 (39.0, 39.3)	-4.0 (-4.7, -3.3)
50-54	3,649.9	15	4.1	122,052.1	281	2.3	30.7 (30.1, 31.2)	34.4 (34.3, 34.6)	-3.7 (-4.3, -3.1)
55-59	5,096.7	37	7.3	107,220.3	374	3.5	26.3 (25.8, 26.8)	29.8 (29.6, 29.9)	-3.5 (-4.0, -3.0)
60-64	6,651.2	61	9.2	98,186.8	547	5.6	22.2 (21.7, 22.6)	25.3 (25.1, 25.4)	-3.1 (-3.5, -2.7)
65-69	7,656.9	124	16.2	90,255.1	765	8.5	18.1 (17.7, 18.4)	20.9 (20.8, 21.0)	-2.8 (-3.2, -2.4)
70-74	8,002.6	197	24.6	66,256.4	1,017	15.3	14.4 (14.1, 14.7)	16.7 (16.6, 16.8)	-2.3 (-2.6, -2.0)
75-79	8,128.1	359	44.2	52,953.9	1,460	27.6	10.9 (10.7, 11.2)	12.8 (12.7, 13.0)	-1.9 (-2.2, -1.6)
80-84	6,287.2	475	75.6	39,938.8	2,202	55.1	8.0 (7.8, 8.3)	9.4 (9.3, 9.5)	-1.4 (-1.7, -1.1)
85-89	3,627.9	529	145.8	25,437.1	2,617	102.9	5.6 (5.4, 5.8)	6.6 (6.5, 6.7)	-1.0 (-1.2, -0.8)

[a] Difference calculated as (type 2 diabetes cohort life expectancy minus type 2 diabetes-free population life expectancy); negative values indicate lower life expectancy in type 2 diabetes cohort.

ESM Table 5

Abridged life table for men and women with type 2 diabetes vs. population of Scotland without type 2 diabetes; SIMD quintile 5 (least deprived), period 2012-2014

	observed data in type 2 diabetes cohort			observed data in type 2 diabetes-free population			estimated life expectancy (95% confidence interval)		
age interval (years)	person-years	deaths	death rate per 1,000 p-y	person-years	deaths	death rate per 1,000 p-y	type 2 diabetes cohort	type 2 diabetes-free population	LE difference[a]
MEN									
40-44	1,453.8	4	2.8	107,031.3	97	0.9	38.7 (37.9, 39.5)	43.2 (43.0, 43.3)	-4.5 (-5.3, -3.7)
45-49	2,942.3	17	5.8	117,537.7	167	1.4	34.2 (33.5, 34.8)	38.4 (38.2, 38.5)	-4.2 (-4.9, -3.5)
50-54	5,082.7	32	6.3	113,050.4	208	1.8	30.1 (29.6, 30.6)	33.6 (33.4, 33.8)	-3.5 (-4.0, -3.0)
55-59	7,407.3	39	5.3	98,282.7	341	3.5	26.0 (25.6, 26.4)	28.9 (28.7, 29.1)	-2.9 (-3.4, -2.4)
60-64	9,381.4	112	11.9	87,298.6	523	6.0	21.6 (21.2, 22.0)	24.4 (24.2, 24.5)	-2.8 (-3.2, -2.4)
65-69	10,859.8	169	15.6	77,699.3	778	10.0	17.8 (17.5, 18.1)	20.0 (19.9, 20.2)	-2.2 (-2.6, -1.8)
70-74	8,913.4	219	24.6	51,644.6	935	18.1	14.0 (13.7, 14.4)	15.9 (15.8, 16.1)	-1.9 (-2.2, -1.6)
75-79	7,993.2	399	49.9	40,364.8	1,298	32.2	10.5 (10.3, 10.8)	12.2 (12.1, 12.3)	-1.7 (-2.0, -1.4)
80-84	5,365.2	436	81.3	26,791.8	1,692	63.2	7.8 (7.6, 8.1)	8.9 (8.8, 9.0)	-1.1 (-1.4, -0.8)
85-89	2,512.0	376	149.7	14,055.0	1,633	116.2	5.6 (5.3, 5.8)	6.3 (6.2, 6.4)	-0.7 (-1.0, -0.4)
WOMEN									
40-44	824.0	2	2.4	115,486.0	71	0.6	40.4 (39.4, 41.4)	45.2 (45.1, 45.4)	-4.8 (-5.8, -3.8)
45-49	1,593.1	6	3.8	127,551.9	137	1.1	35.8 (35.0, 36.7)	40.4 (40.2, 40.5)	-4.6 (-5.4, -3.8)
50-54	2,721.8	12	4.4	121,466.2	234	1.9	31.5 (30.8, 32.1)	35.6 (35.4, 35.7)	-4.1 (-4.8, -3.4)
55-59	3,925.6	25	6.4	106,223.4	308	2.9	27.1 (26.6, 27.7)	30.9 (30.7, 31.0)	-3.8 (-4.4, -3.2)
60-64	5,063.7	45	8.9	96,477.3	430	4.5	22.9 (22.4, 23.4)	26.3 (26.2, 26.4)	-3.4 (-3.9, -2.9)
65-69	6,549.7	88	13.4	88,685.3	642	7.2	18.9 (18.4, 19.3)	21.8 (21.7, 22.0)	-2.9 (-3.3, -2.5)
70-74	6,346.5	140	22.1	63,990.6	830	13.0	15.0 (14.6, 15.4)	17.6 (17.4, 17.7)	-2.6 (-3.0, -2.2)
75-79	6,470.5	264	40.8	52,595.5	1,244	23.7	11.4 (11.1, 11.8)	13.6 (13.4, 13.7)	-2.2 (-2.5, -1.9)
80-84	5,294.5	405	76.5	39,573.5	1,808	45.7	8.5 (8.2, 8.8)	9.9 (9.8, 10.1)	-1.4 (-1.7, -1.1)
85-89	2,933.7	371	126.5	25,341.3	2,410	95.1	6.3 (6.0, 6.6)	6.9 (6.8, 7.0)	-0.6 (-0.9, -0.3)

[a] Difference calculated as (type 2 diabetes cohort life expectancy minus type 2 diabetes-free population life expectancy); negative values indicate lower life expectancy in type 2 diabetes cohort.
